# Dataset on ecological fiscal transfers and municipal protected areas in the state of Minas Gerais, Brazil

**DOI:** 10.1016/j.dib.2019.104601

**Published:** 2019-10-03

**Authors:** Felipe Luiz Lima de Paulo, Pedro Jorge Sobral Camões

**Affiliations:** a Universidade Federal Rural de Pernambuco, Brazil; b Universidade do Minho, Portugal

**Keywords:** Ecological fiscal transfers, Municipal protected area, Minas Gerais, Brazil

## Abstract

This dataset was collected in the state government of Minas Gerais, Brazil (*Instituto Estadual de Florestas*), regulatory deliberation 86/2005 of the state of Minas Gerais, law 12040/1995 of the state of Minas Gerais, law 18030/2009 of the state of Minas Gerais, Brazilian law 9985/2000, and some laws/decrees that created municipal protected areas. The data was used to analyze the influence of the ecological fiscal transfers (EFT) in the policy-making process of adopting protected areas by municipal governments in the state of Minas Gerais. It has the potential to be reused in other studies to analyze the EFT at the local level. The related research article that uses this database was published under the title “Ecological Fiscal Transfers for Biodiversity Conservation Policy: A Transaction Costs Analysis of Minas Gerais, Brazil” [1].

**Table:** Specifications Table

Subject	Economics, Econometrics and Finance (General)
Specific subject area	Ecological Economics
Type of data	Table
How data were acquired	This dataset was collected in the state government of Minas Gerais, Brazil (*Instituto Estadual de Florestas*), under the regulatory deliberation 86/2005 of the state of Minas Gerais, law 12,040/1995 of the state of Minas Gerais, law 18030/2009 of the state of Minas Gerais, and Brazilian law 9,985/2000. Also, some data was also complemented with the laws/decrees that created protected areas.
Data format	Raw and Analyzed
Parameters for data collection	Due to the scarcity of data to study ecological fiscal transfers, these datasets have the potential for academics and practitioners interested in ecological fiscal transfers for biodiversity conservation policies at the local level.
Description of data collection	The data of the municipal protected area created from 1966 to 2013, including the group (sustainable or integral) and categories adopted in the State of Minas Gerais, was collected in the state of Minas Gerais under the law on access to public information (law 12,527/2011). Some data was also complemented with the laws/decrees that created municipal protected areas. The information related to the implementation of the quality index was collected in the regulatory deliberation 86/2005 of the state of Minas Gerais. The area of the protected area over the total of the territory of the municipality in hectares (share of the area) was collected in the state of Minas Gerais under the law on access to public information. Also, the municipal laws/decrees and the Brazilian Institute of Geography and Statistics (IBGE) complemented the information. The conservation factor for each category of the protected area was collected in the law 18,030/2009. The information related to EFT adoption was collected in the law 12,040/1995. The duration until the adoption of protected area (duration) and the year of the adoption of the protected area (event) was collected in the state of Minas Gerais under the law on access to public information (law 12,527/2011).
Data source location	State of Minas Gerais, Brazil
Data accessibility	Data is supplied with the paper
Related research article	Author's name: Felipe Luiz Lima de Paulo; Pedro Jorge Sobral Camões Title: Ecological Fiscal Transfers for Biodiversity Conservation Policy: A Transaction Costs Analysis of Minas Gerais, Brazil Journal: Ecological Economics DOI: https://doi.org/10.1016/j.ecolecon.2019.106425

**Table:** 

**Value of the data** • It has the potential to be reused in other studies to analyze the EFT at the local level, as well as in studies related to biodiversity conservation policies at the local level. • Academics and practitioners interested in biodiversity conservation policies at the local level and ecological fiscal transfers. • These data can be helpful to academics and practitioners to study municipal protected areas classified by categories which varies according to the level of land-use restrictions.

## 1 Data

The dataset contains raw and analyzed data of protected areas (PAs) created by municipal governments in the state of Minas Gerais from 1966 to 2013. The data files (do-files) were deposited at Mendeley (http://doi.org/10.17632/sgfhcz98ck.1). The data were gathered from the State Government of Minas Gerais under the Brazilian law on access to public information (law 12,527/2011). Also, additional data were collected from laws/decrees of the municipal governments that created PAs.

Protected areas are classified into five categories: municipal park (PM), environmental protected area (APA), biological reserve (REBIO), natural monuments (MONA), municipal forest (FLOMA). The categories of protected areas are classified into two groups: sustainable (APA, FLOMA) and integral protection (REBIO, PM, MONA). The name and the measurement of the variables used in the study are described in Table 1.

**Table 1:** Variables.

Variable name	Measurement
Duration	Duration until the adoption of protected area
Event	1 in case of protected area adoption, 0 otherwise
EFT	1 for the years after EFT adoption in 1996, 0 otherwise
Conservation Factor	Conservation factor for each protected area category
Share of Area	Area of the protected areas over the total area of the territory of the municipality (ha)
Quality Index	1 after the implementation of the quality index in 2005, 0 otherwise
APA Instituto Estadual de Florestas (MG)	1 for the years after APA category adopted, 0 otherwise
PM Instituto Estadual de Florestas (MG)	1 for the years after PM category adopted, 0 otherwise
REBIO Instituto Estadual de Florestas (MG)	1 for the years after REBIO category adopted, 0 otherwise
MONA Instituto Estadual de Florestas (MG)	1 for the years after MONA category adopted, 0 otherwise
FLOMA Instituto Estadual de Florestas (MG)	1 for the years after FLOMA category adopted, 0 otherwise
SNUC Instituto Estadual de Florestas (MG)	1 for the years after SNUC adopted, 0 otherwise

## 2 Experimental design, materials, and methods

To perform the analysis to understand the influence of EFT in the policy-making process of adopting PAs by municipal governments [1], a comprehensive descriptive analysis of the data was performed. First, was analyzed the years until the adoption of protected areas, and must of the protected areas were created by municipal governments between the 32nd and 37th years (Table 2). Also, most of the PAs adopted between the 32nd and 37nd years belonged to the sustainable group (see Table 3). The time that increased the adoption of PAs between the 32nd and 37th overlapped with the introduction of EFT (see Table 4) as well as with the introduction of the national system of protected areas (Table 5, Table 6), a national policy that shaped many aspects concerning PAs at the local level. After EFT implementation and before the creation of the national system of protected areas, 44 PAs were created, while after the introduction of both policy tools, 122 PAs were created. The municipal governments were more attracted to create less restrictive protected areas after EFT implementation, that is, PAs with 0.5 weight to conservation factor (see Table 7, Table 8). However, after the adoption of the quality index by the state government, the creation of PAs decreased (see Table 9).

**Table 2:** Years until the adoption of protected area.

Years until PA adoption	Freq.	Percent	Cum.
0	1	0.50	0.50
1	1	0.50	1.01
10	3	1.51	2.51
11	1	0.50	3.02
12	2	1.01	4.02
13	2	1.01	5.03
14	1	0.50	5.53
16	5	2.51	8.04
18	1	0.50	8.54
21	1	0.50	9.05
22	2	1.01	10.05
23	1	0.50	10.55
24	2	1.01	11.56
25	5	2.51	14.07
26	2	1.01	15.08
28	3	1.51	16.58
30	1	0.50	17.09
31	8	4.02	21.11
32	18	9.05	30.15
33	17	8.54	38.69
34	10	5.03	43.72
35	45	22.61	66.33
36	36	18.09	84.42
37	17	8.54	92.96
38	3	1.51	94.47
39	1	0.50	94.97
41	1	0.50	95.48
42	2	1.01	96.48
43	1	0.50	96.98
46	1	0.50	97.49
47	5	2.51	100.00
Total	199	100.00

**Table 3:** Year of the creation of the protected area per group.

Year of the creation of the PA	The group of municipal PA		
	Integral	Sustain	Total
1966	0	1	1
1967	1	0	1
1976	3	0	3
1977	1	0	1
1978	2	0	2
1979	2	0	2
1980	1	0	1
1982	5	0	5
1984	1	0	1
1987	1	0	1
1988	2	0	2
1989	1	0	1
1990	2	0	2
1991	3	2	5
1992	1	1	2
1994	1	2	3
1996	1	0	1
1997	3	5	8
1998	7	11	18
1999	2	15	17
2000	1	9	10
2001	1	44	45
2002	5	31	36
2003	1	16	17
2004	0	3	3
2005	0	1	1
2007	1	0	1
2008	2	0	2
2009	1	0	1
2012	0	1	1
2013	5	0	5
Total	57	142	199

**Table 4:** Year of the creation of the protected area before and after EFT adoption.

Year of the creation of the PA	Ecological Fiscal Transfers		
	0	1	Total
1966	1	0	1
1967	1	0	1
1976	3	0	3
1977	1	0	1
1978	2	0	2
1979	2	0	2
1980	1	0	1
1982	5	0	5
1984	1	0	1
1987	1	0	1
1988	2	0	2
1989	1	0	1
1990	2	0	2
1991	5	0	5
1992	2	0	2
1994	3	0	3
1996	0	1	1
1997	0	8	8
1998	0	18	18
1999	0	17	17
2000	0	10	10
2001	0	45	45
2002	0	36	36
2003	0	17	17
2004	0	3	3
2005	0	1	1
2007	0	1	1
2008	0	2	2
2009	0	1	1
2012	0	1	1
2013	0	5	5
Total	33	166	199

**Table 5:** Year of the creation of the protected area before and after the introduction of the national system of protected areas.

Year of the creation of the PA	National System of Protected Areas adopted		
	0	1	Total
1966	1	0	1
1967	1	0	1
1976	3	0	3
1977	1	0	1
1978	2	0	2
1979	2	0	2
1980	1	0	1
1982	5	0	5
1984	1	0	1
1987	1	0	1
1988	2	0	2
1989	1	0	1
1990	2	0	2
1991	5	0	5
1992	2	0	2
1994	3	0	3
1996	1	0	1
1997	8	0	8
1998	18	0	18
1999	17	0	17
2000	0	10	10
2001	0	45	45
2002	0	36	36
2003	0	17	17
2004	0	3	3
2005	0	1	1
2007	0	1	1
2008	0	2	2
2009	0	1	1
2012	0	1	1
2013	0	5	5
Total	77	122	199

**Table 6:** Protected areas adopted before and after EFT adoption and National System of Protected Area adoption.

Ecological Fiscal adopted Transfers	National System of Protected Areas		
	0	1	Total
0	33	0	33
1	44	122	166
Total	77	122	199

**Table 7:** Protected area adopted before and after EFT adoption by conservation factor of the protected area.

Ecological Fiscal Transfers	Conservation Factor of the PA						
	0	.25	.5	.7	.9	1	Total
0	33	0	0	0	0	0	33
1	0	1	134	1	14	16	166
Total	33	1	134	1	14	16	199

**Table 8:** Number of protected area created by year and grouped by conservation factor of the protected area.

Year of The Creation of the PA	Conservation Factor of the PA						
	0	.25	.5	.7	.9	1	Total
1966	1	0	0	0	0	0	1
1967	1	0	0	0	0	0	1
1976	3	0	0	0	0	0	3
1977	1	0	0	0	0	0	1
1978	2	0	0	0	0	0	2
1979	2	0	0	0	0	0	2
1980	1	0	0	0	0	0	1
1982	5	0	0	0	0	0	5
1984	1	0	0	0	0	0	1
1987	1	0	0	0	0	0	1
1988	2	0	0	0	0	0	2
1989	1	0	0	0	0	0	1
1990	2	0	0	0	0	0	2
1991	5	0	0	0	0	0	5
1992	2	0	0	0	0	0	2
1994	3	0	0	0	0	0	3
1996	0	0	0	0	1	0	1
1997	0	0	5	0	3	0	8
1998	0	0	11	0	5	2	18
1999	0	0	15	0	2	0	17
2000	0	0	9	0	1	0	10
2001	0	0	44	0	1	0	45
2002	0	0	30	1	0	5	36
2003	0	0	16	0	0	1	17
2004	0	0	3	0	0	0	3
2005	0	0	1	0	0	0	1
2007	0	0	0	0	1	0	1
2008	0	0	0	0	0	2	2
2009	0	0	0	0	0	1	1
2012	0	1	0	0	0	0	1
2013	0	0	0	0	0	5	5
Total	33	1	134	1	14	16	199

**Table 9:** Number of protected area created before and after EFT adoption and before and after quality index adopted.

Ecological Fiscal Transfers	Quality Index adopted		
	0	1	Total
0	33	0	33
1	155	11	166
Total	188	11	199
